# Experimental Investigation and Optimization of Rough EDM of High-Thermal-Conductivity Tool Steel with a Thin-Walled Electrode

**DOI:** 10.3390/ma16010302

**Published:** 2022-12-28

**Authors:** Dorota Oniszczuk-Świercz, Rafał Świercz, Adrian Kopytowski, Rafał Nowicki

**Affiliations:** Institute of Manufacturing Technology, Warsaw University of Technology, 02-524 Warsaw, Poland

**Keywords:** thin-walled electrode, EDM, optimization, high thermal conductive tool steel (HTCS), analysis of variance (ANOVA), derringer’s function

## Abstract

The industrial application of electrical discharge machining (EDM) for manufacturing injection molding, in many cases, requires forming depth cavities with high length-to-width ratios, which is quite challenging. During slot EDM with thin-walled electrodes, short-circuits and arcing discharges occur, as a result of low efficiency in removing debris and bubble gas from the gap. Furthermore, unstable discharges can cause increases in tool wear and shape deviation of the machined parts. In order to characterize the influence of the type of electrode material and EDM parameters on the deep slot machining of high-thermal-conductivity tool steel (HTCS), experimental studies were conducted. An analytical and experimental investigation is carried out on the influence of EDM parameters on discharge current and pulse-on-time on the tool wear (TW), surface roughness (Ra), slot width (S)—dimension of the cavity, and material removal rate (MRR). The analyses of the EDS spectrum of the electrode indicate the occurrence of the additional carbon layer on the electrode. Carbon deposition on the anode surface can provide an additional thermal barrier that reduces electrode wear in the case of the copper electrode but for graphite electrodes, uneven deposition of carbon on the electrode leads to unstable discharges and leads to increase tool wear. The response surface methodology (RSM) was used to build empirical models of the influence of the discharge current *I* and pulse-on-time *t*_on_ on Ra, S, TW, and MRR. Analysis of variance (ANOVA) was used to establish the statistical significance parameters. The calculated contribution indicated that the discharge current had the most influence (over 70%) on the Ra, S, TW, and MRR, followed by the discharge time. Multicriteria optimization with Derringer’s function was then used to minimize the surface roughness, slot width, and TW, while maximizing MRR. A validation test confirms that the maximal error between the predicted and obtained values did not exceed 7%.

## 1. Introduction

In electrical discharge machining (EDM), a series of electrical discharges occur in the gap between an anode and cathode immersed in the dielectric. Thermal energy induced by discharge causes local melting and evaporation of the workpiece and tool electrode. In the place of electrical discharge, the crater is formed. In many cases an industrial application of EDM requires manufacturing of cavities with thin-walled electrodes having a high ratio of electrode length to thickness. In this case, the key role in slot EDM is the efficiency of removing debris and bubble gas from the gap. Die-sinking EDM with monolithic electrodes uses the additional movements of the head with the electrode, where the oscillation of the head increases the thickness of the gap during machining, to flush away the debris by the fresh dielectric. The presence of particles of eroded material in the gap may also lead to a local increase in the number of electrical discharges caused by a decrease in dielectric resistance. Effective flushing erosion products outside the gap ensure stable discharges. Murray et al. [1] indicated that erosion products change the resistance of the dielectric in the gap. The concentration of debris can lead to induce discharge at the same location. Moreover, the ineffective flushing of debris from the gap provides short-circuits and arcing [2]. The occurrence of unstable discharges increases tool wear, leading to significant deviations in the shape of the manufactured geometry [3,4,5,6]. Furthermore, unstable discharges have a strong influence on surface-layer properties and surface roughness after EDM. Many studies are considered improving surface layer properties after EDM [7,8,9,10,11]. Conducted research in this field has focused on using additional technologies to improve surface layer properties. One of the most used is a powder mixed EDM [12,13,14], applying coatings [15,16,17,18], and non-conventional machining [19,20,21,22]. Nevertheless using additional technologies significantly increases the manufacturing costs. It is therefore important to select the optimal parameters of electrical discharge machining for the obtain desired results.

EDM is characterized by its complexity, resulting both from the material removal physics itself and from the machining conditions affecting its stability [23,24,25,26]. Roy et al. [27] indicated that considering the electric field, discharges in the gap could be divided into individual cases. In the first stage, discharges occur in the place where the gap is the smallest. Next, discharges can occur on the single erosion produced. Finally, discharges will appear in the place of concentration of debris. The research carried out by the authors of [28] shows that the occurrence of conductive particles in the gap leads to the multiplication of discharges on particles, which significantly affects the EDM process. Kitamura et al. [29] indicated that the stability of EDM also depends on the gas bubbles generated by electrical discharges, which occupy the gap. It was found that bubbles fill more than 70% of the gap. The probability of discharges is highest at the boundary of bubbles, where the debris particles are concentrated. Wang et al. [30] showed that the bubble expansion movement is the primary mode of debris removal outside the machining gap. Research carried out by Yue et al. [31] shows that the collision of the vaporized material of the electrode and workpiece has a significant impact on the process. For electrodes with lower bowling points observed an increase in removing melted material from the workpiece. Research carried out by Kunieda et al. [32] showed that the plasma channel induced by an electrical discharge not only causes melting and evaporation of material from the anode and cathode but also leads to the dissociation of carbon from the oil dielectric. As a result, the carbon layer on the copper anode decreases the wear of the anode. Similar results were presented by Maradia et al. [33] Carbon deposition on the anode surface can provide an additional thermal barrier that reduces electrode wear. Murray et al. [34] pointed out that in the case of manufacturing micro slots, the deposited layer protects the side wall from secondary discharges. Furthermore, the average thickness of the carbon layer increases with the growth of discharge energy and decreases with the frequency of discharges. Flano et al. [35] analyzed the influence of tool geometry on the productivity of manufacturing depth slot with multi holed electrodes. The presented results indicated that the modified shape of the electrode by adding the pockets on its bottom face increases the efficiency of debris evacuation and stabilizes the discharges. The research conducted by Ayesta et al. [36] showed that the efficiency of removing erosion products depends on the shape of the cavity. For inclined slots, the effect of gravity facilitates the removal of debris from the gap. Klocke et al. [37] investigate the influence of the used material of electrode on EDM productivity. The presented results indicate that bronze infiltrated graphite can replace a copper-tungsten electrode during the machining of Cemented Carbides. D’Urso et al. [38] indicate that the chosen material electrode may be based on a sustainability index which includes tool wear and energy consumption. Furthermore, the final choice of material electrode should include the obtained precision of manufacturing parts using different types of material electrodes. Mathai et al. [39] analyzed the influence of using cooper and graphite electrodes in die-sinking EDM of Ti6Al4V. Presented results show that using the graphite electrode is possible to increase the material removal rate, however, the copper electrodes are better choices when considering the wear of the electrode and the final surface quality of manufacturing parts. 

The complexity of physical phenomena occurring in the EDM causes difficulty in precision describing of influence of process parameters on productivity. Proper selection of machining parameters plays a key role in the process. Previous studies indicate that the most significant parameters affected by the EDM are discharge current and pulse-on-time [40]. For constant discharge voltage, these parameters represent the discharge energy. The time interval between the discharges significantly affects the MRR. For short time intervals toff, the frequency of discharges grows, leading to an increase in MRR. However, very short break times lead to disturbed conditions in the gap. The presence of erosion products in the gap causes a local reduction in the dielectric resistance, resulting in uneven discharges occurring along the cavity. This can contribute to obtaining shape deviations in the manufacturing part and uneven wear of the tool electrode.

In industrial applications, EDM predictive models have been built on empirical data based on response surface methodology [41,42,43], artificial neural networks or deep machine learning [44,45,46,47,48,49]. Furthermore, the optimal selection of EDM parameters, especially for new materials like high-thermal-conductivity tool steel (HTCS) requires conducting experimental studies. In EDM technology appropriate selection of parameters and types of electrodes for various manufactured materials has a significant impact on its productivity. The research carried out so far indicates that the selection of the electrode material should be provided in the conducted experimental studies considering the EDM parameters and type of processing material. One of the new materials which have a range of industrial applications is HTCS. Due to its properties, high thermal conductivity (66 W/mK), and hardness (52 HRC) has wide application in dies for plastic injection molds or casting. The construction of injection molds, in many cases, involves depth cavities with high length to thickness ratios. This is difficult to perform using conventional manufacturing technology. One of the technologies which allow for the manufacture of this type of cavity is EDM. However, manufacturing deep slots with thin electrodes is a challenging task also to EDM. In the published literature, few studies have focused on high depth slot sinking EDM [50,51,52,53,54]. The main research problem in manufacturing deep slot with thin-walled electrode is unstable discharges in the face of the electrode caused by the inefficient evacuation of debris and bubble gas from the gap. The published literature indicates that few studies have reported on the manufacturing deep slot with thin-walled electrode. However, analyses of the influence of discharge current and pulse-on-time on HTCS with thin-walled electrodes have not been investigated.

A novel of this work is the analysis and discussion of the influence of EDM parameters during deep slot machining of new material HTCS with two different materials of electrode copper and graphite. The thermal energy induced by discharge causes local melting and evaporation of the workpiece and tool electrode. Due to the complex the physics of removal mechanism in EDM it is difficult to predict the influence of the process parameter on the machining indicators such as surface roughness, tool wear, geometric accuracy, and material removal rate. Therefore, in the case of developing EDM technologies for new materials such as HTCS, it is necessary to conduct experimental research that will allow determining the relationship between the process parameters and technological indicators. In addition, in the case of treatment with thin-walled electrodes, difficulties in evacuating the erosion products from the gap occur which leads to short-circuiting discharges. 

Industrial application of conducted research required building prediction models of the process. Opposing requirements: minimize surface roughness, slot width, and TW, while maximizing the material removal rate indicates that this is a quite challenging task. Therefore, the main goal of this study was to determine the impact of the dis-charge current and pulse-on-time, on the surface roughness, the tool wear (TW), surface roughness (Ra), slot width (S)—dimension of the cavity, and material removal rate (MRR). To achieve this goal, prediction models of EDM with a thin-walled electrode of HTCS with response surface methodology (RSM) were developed. In order to characterize the influence of the type of electrode material and EDM parameters on the deep slot machining of HTCS, experimental studies were conducted for copper and graphite electrode.

## 2. Materials and Methods

Conducted experimental research aims to investigate the influence of discharge current and pulse-on-time on rough-slot machining in high-thermal-conductivity tool steel. Due to its properties, high thermal conductivity (66 W/mK), and hardness (52 HRC) has wide application in dies for plastic injection molds or casting. The chemical composition of Rovalma HTCS 150 (mass%) is presented in Table 1.

**Figure 1:**
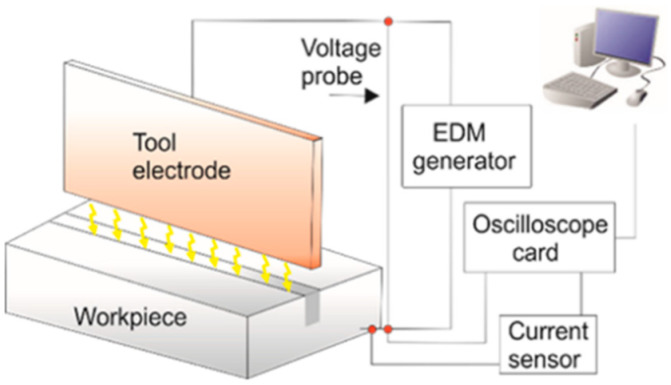
Scheme of research set-up: measurement circuit of voltage and current.

The research was carried out on the Charmilles Form 2LC ZNC machine (GF Solutions, Bienne, Switzerland). A block of material with dimensions of 200 × 200 × 100 mm was manufactured from copper, a graphite electrode (POCO EDM 3, Entegris, Inc., Billerica, MA, USA) in the hydrocarbon-based dielectric. The cross section of electrodes was a rectangle with dimensions of 100 mm × 1 mm, respectively, in length and width. Each slot was machining to the depth of 10 mm with a new electrode. A scheme of research setup is presented in Figure 1. The physical properties of electrodes are presented in Table 2.

**Figure 2:**
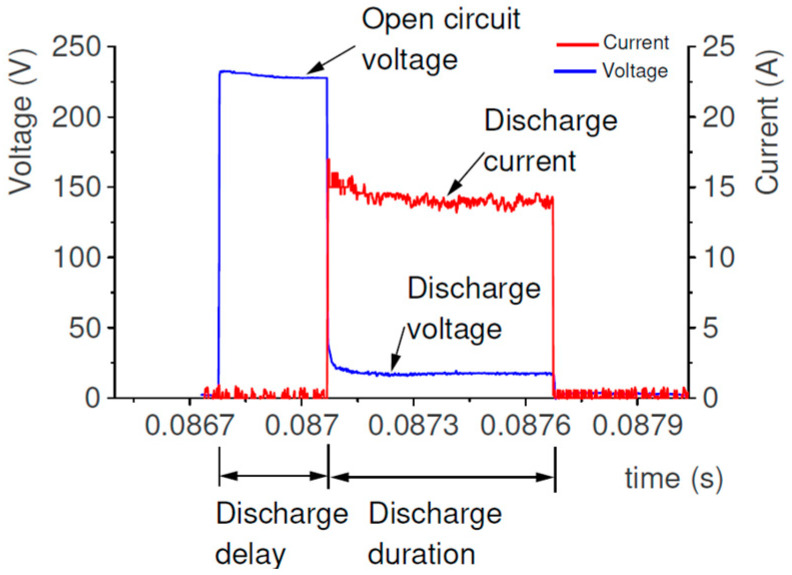
The registered waveform of discharge current and voltage.

The initial research and literature review indicate that the material removal mechanism in EDM mainly depends on the discharge current and pulse-on-time. In the first stage of conducted experimental research, the range of stable discharge was established. Measurement of the current and voltage waveforms was carried out using a NI5133 oscilloscope card (National Instruments, Austin, TX, USA). The voltage and current measurements were carried out using a probe Tektronix and a non-inductive current sensor, respectively. During the measuring, the sampling rate was established as equal to 100 MS/s, for two channels. DIAdem software (National Instruments) was used to analyze recorded data. The typically recorded waveforms during slot die-sinking EDM in HTCS with a copper electrode are shown in Figure 2. 

The initiation of discharge occurred when the intensity of the voltage field overcame the dielectric resistance in the gap between the two electrodes. The open-circuit voltage drops to discharge voltage and the intensity increases to discharge current. After exceeding the dielectric breakdown strength, a plasma channel is formed. Around the plasma channel, a bubble gas is created, which is filled with ions and parts of melted material of the workpiece and the electrode. At the end of the discharge, the bubble gas and plasma channel implosively collapse. The melted material is thrown away to the gap and rapidly cooled down by the dielectric. The molten material is re-solidified into hundreds of spherical particles. Depending on the discharge energy, the size of debris varies and can reach several micrometers [1]. The debris and bubble gas are removed from the gap by the flushing dielectric. The conditions in the gap stabilize during a time interval. Another discharge takes place in a random place. The preliminary manufacturing test of the EDM slot in HTCS was conducted to find a stable range of machining parameters (i.e., discharge current and pulse-on-time). Analysis of the recorded voltage and current waveforms for over fifty experimental tests indicates that, with the highest discharge currents and discharge pulse-time-on along with the short time interval (below 0.3 of value pulse-time-on), short-circuits and arcing discharges occurred, in most cases. The analysis of voltage and current waveforms allowed to select the stable parameters in the EDM process for rough slotting machining (Table 3).

Experimental studies of influence discharge current and pulse-on-time: on surface roughness Ra, slot width S, MRR, and TW during slot machining of HTCS were conducted using the Hartley experiment design methodology, with 5 levels and 2 parameters. Table 4 shows the levels of machining parameters used in the experimental design.

**Figure 3:**
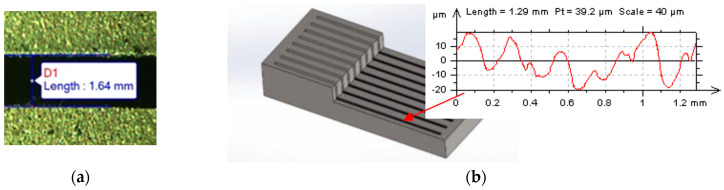
Scheme of measuring: (**a**) slot width on the optical microscope; (**b**) surface roughness.

Each manufacturing surface was measured three times. The slot width S—dimension of the cavity was measured with an optical microscope XJA-6A (Figure 3a). Surface roughness was measured on the bottom of the manufacturing slot. The workpiece was cut by Wire EDM to ensure the contact of the measuring head with the surface (Figure 3b). Measurement was performed using a Taylor-Hobson FORM TALYSURF Series 2 scan profilometer. EDS spectrum of electrode surface was investigated by the JEOL JCM-7000 (NeoScope).

Material removal rate was calculated based on the measured each sample: slot width, length and depth (which define the volume of material removed from the workpiece) divided by machining time:

(1) $$\mathrm{MRR}=\frac{S\;l\;h}{\;\Delta t}\left[\frac{{\mathrm{mm}}^{3}}{\min}\right],$$

where

*S*—slot width after processing,
*h*—slot depth after processing,
*l*—slot length after processing,
∆*t*—a time of manufacturing.

Each electrode was mounted in the EROWA ITS chuck in the EDM head and, after manufacturing the slot, the tool wear of the electrode was measured using the coordinate measuring machine (CMM) Carl Zeiss Vista. Each electrode was mounted in the EROWA ITS chuck on the CMM table. The software Calypso Carl Zeiss (cooperating with CMM) measurement strategy for the top surface (face plane ) of the electrode was determined. The measurement was performed using a raster—each of the surfaces was measured by means of 50 measurement points. The obtained results were used to determine the mean plane. In the next step, the shortening of the working electrode was calculated by determining the distance between the face plane of the new electrode and the electrode that was used to manufacturing the slot.

Response surface methodology (RSM) was used to develop the predictive models of influence discharge current and pulse-on-time on the surface roughness Ra, sloth width S, MRR, and TW. The response surface was estimated according to the following equation:

(2) $$Y=f\;(I,t_{\mathrm{on}})\;\pm \varepsilon ,$$

where *Y* is the investigated response (surface roughness Ra, sloth width, MRR, and TW), *f* is the polynomial function of the second degree, *ε* is the experimental error, and *I* (the discharge current), and *t*_on_ (the pulse-on-time) are independent parameters.

The industrial implementation of the developed models should take into account the optimization of EDM parameters. Roughing machining should use parameters that allow for the maximal MRR to be achieved with minimal slot width S, TW, and surface roughness. In the case of EDM, achieving these four goals simultaneously is conflicting in nature. For this reason, multi-response optimization was performed with Derringer’s desirability approach. The desirability function can be calculated by the following equation:

(3) $$D={\left(d_{1}\times d_{2}\times \ldots \ldots \times d_{n}\right)}^{1/n}={\left(\prod_{i=1}^{n}d_{i}\right)}^{\frac{1}{n}},$$

where *n* is the number of functions which are taking account in the optimization.

In this study, the Derringer’s function was developed for each function $d_{i}({\hat{y}}_{i})$ (surface roughness Ra, slot width S, MRR, and TW). Depending on the criteria of optimization, some responses should be maximized or minimized. For maximized response function $d_{i}({\hat{y}}_{i})$ following equation was calculated:

(4) $$d_{i}\left({\hat{y}}_{i}\right)=\{\;\begin{array}{c} 0\\ {\left(\frac{{\hat{y}}_{i}-Li}{U_{i}-Li}\right)}^{s}\\ 1 \end{array},\;\begin{array}{c} {\hat{y}}_{i}<L_{i}\\ L_{i}\leq {\hat{y}}_{i}\leq U_{i}\\ {\hat{y}}_{i}>U_{i} \end{array}$$

For maximized response function $d_{i}({\hat{y}}_{i})$ following equation was calculated:

(5) $$d_{i}\left({\hat{y}}_{i}\right)=\{\;\begin{array}{c} 1\\ {\left(\frac{U_{i}-{\hat{y}}_{i}}{U_{i}-L_{i}}\right)}^{t}\\ 0 \end{array},\;\begin{array}{c} {\hat{y}}_{i}<L_{i}\\ L_{i}\leq {\hat{y}}_{i}\leq U_{i}\\ {\hat{y}}_{i}>U_{i} \end{array},$$

**Figure 4:**
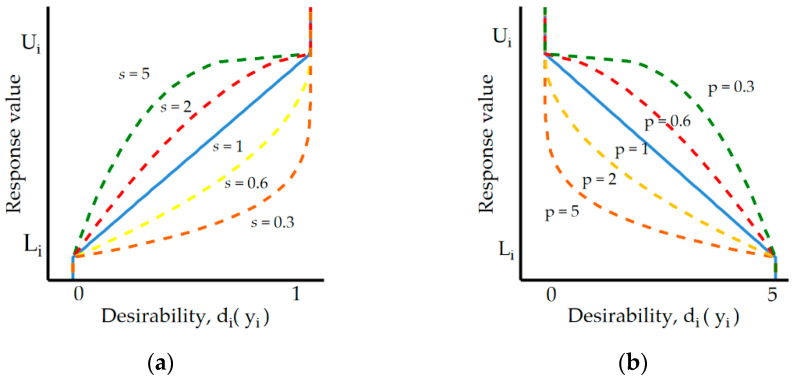
Derringer’s functions for criteria optimization: (**a**) maximized; (**b**) minimized.

Derringer’s desirability optimization methodology allows consideration of the importance of s and *p* for each of the response function that is used in multi-response optimization (Figure 4). The adoption of low values of *t* and *s* causes the desirability function reaches a near target value (one) for a wide range of responses. For large values *p* (for minimum) and s (for maximum), desirability is low if do not reach a near target value.

In the present study, we adopted the following criteria of optimization. Importance *p* = 0.3 for response function Ra, S, TW (goal of minimized) and for MRR importance s = 5 (goal of maximized).

A confirmation test was performed to check the error between the predicted results of optimization and observed values. Three additional samples were manufactured using the established optimal EDM parameters for the rough slotting of HTCS 150.

## 3. Results and Discussion

In the present study, the analysis of the material removal process during the rough-slot EDM in HTCS with a thin-walled electrode was conducted. The research was divided into two stages. In the first stage, an analysis of tool wear electrodes (copper and graphite) was provided. In the second part of the research, the regression function was developed to describe and predict the influence of discharge current and pulse-on-time on the surface roughness Ra, slot width S, MRR, and TW. Finally, multi response optimization with the Derringers function was performed to minimize surface roughness, slot width, and TW, while maximizing the material removal rate. The results of each part of the conducted research were presented and discussed separately.

### 3.1. Analysis of Tool Wear Electrode

**Figure 5:**
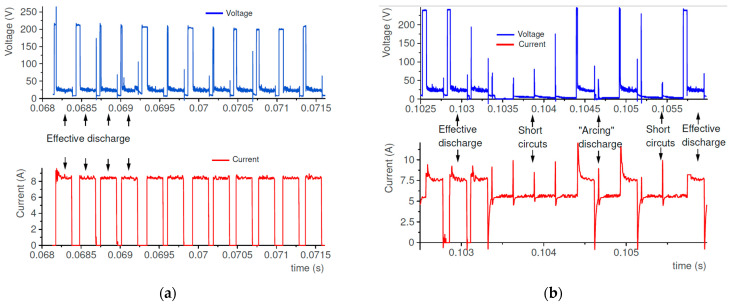
Current and voltage waveforms recorded during EDM for: (**a**) cooper; (**b**) graphite electrode.

Experimental tests using two different types of electrode materials—copper and graphite—were conducted. Analysis of current and voltage waveforms indicated that, in the case of manufacturing high-thermal-conductivity tool, the stability of electrical discharges was significantly different (Figure 5). In the case of slot EDM with the copper electrode, in most investigated range of parameters, stability and repeatability of discharges were observed (Figure 5a). However, in the case of using the graphite electrode for the same EDM parameters: discharge current, discharge voltage, pulse-on-time, time interval, short-circuits, and arcing discharges were observed (Figure 5b).

**Figure 6:**
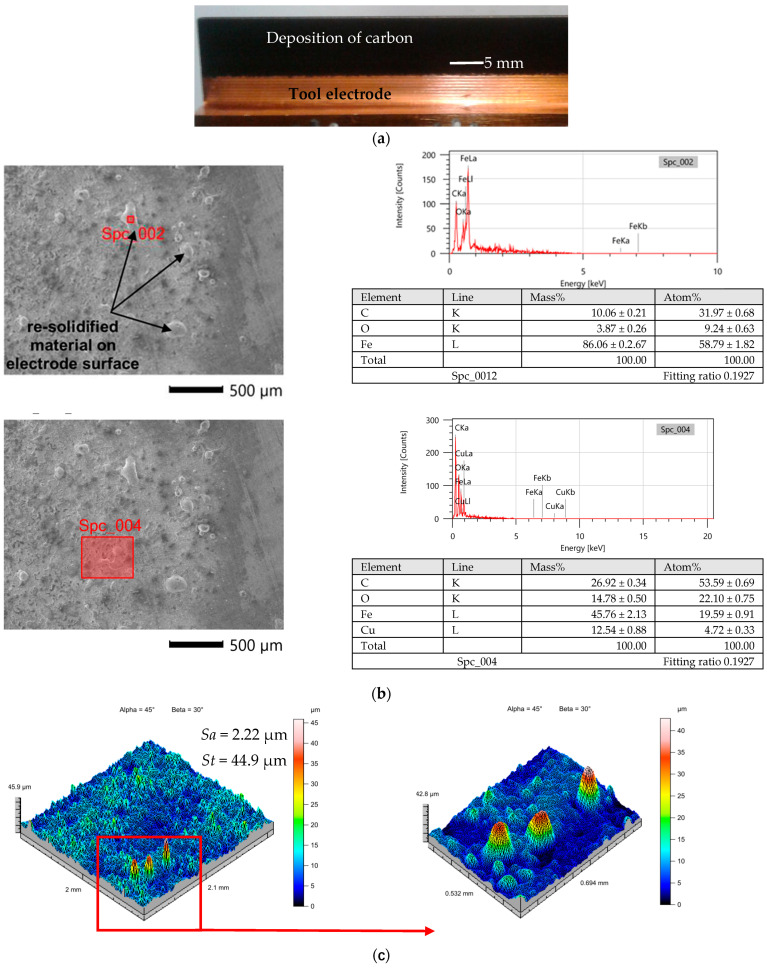
Analysis of the surface properties of the copper electrode: (**a**) an electrode after manufacturing slot; (**b**) EDS spectrum of the surface of the copper electrode, (**c**) measured topography of the surface with marked peaks.

The EDM plasma channel induced by an electrical discharge has a locally high temperature, which causes not only melting and evaporation of material from the anode and cathode but also leads to the dissociation of carbon from the oil dielectric [32]. The deposition of carbon on the anode surface can provide an additional thermal barrier that reduces electrode wear [55]. In the case of slot machining of HTCS, a deposition structure on the electrode (Figure 6a) was observed. The EDS spectrum of the sidewall of the electrode confirms the occurrence of the carbon layer and re-solidified material structure on the sidewall of the electrode (Figure 6b). The EDS spectrum indicates that the new sidewall structure on the electrode has the composition of carbon, copper, oxide and iron elements. Hoverer the peaks of re-solidified material in composition have mostly iron elements (Figure 6b) and have a high even to 40 µm (Figure 6c). The presence of peaks of re-solidified material on the sidewall decrease the distance in the gap between the surface of side wall electrode and surface of slot cavity. Furthermore, this consequently can lead to an increasing number of discharges between of walls of the electrode and cavity and provide to increase in the width of the manufacturing slot.

In die-sinking EDM most discharges occur between the top face of the electrode and the material. Results of the measure of tool wear indicate that for stable discharges, the electrode wears evenly over the entire top face of the surface. 

**Figure 7:**
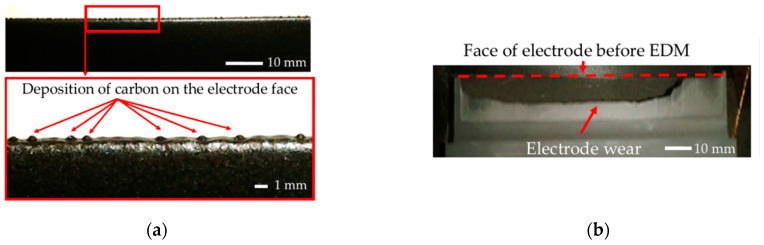
Graphite electrode: (**a**) face of tool electrode after two minutes of work; (**b**) uneven electrode wear after processing slot.

In the second stage of the test, slot cavities in high thermal conductive tool steel were manufactured with a graphite electrode (Figure 7a). At the beginning of manufacturing, the tool wear reached negative values. Observation of the electrode face after two minutes of work showed a local increase of material on the electrode (Figure 7b), leading to a change in the gap size. The carbon deposition of dielectric cause the diffusion of carbon to the graphite electrode. The research conducted by Kunieda [56] and Klocke et al. [37] indicated that, in the case of manufacturing tool steel, properly controlling the carbon diffusion can lead to near-zero electrode wear. However, in the analyzed case of rough-slot EDM of HTCS, the diffusion of carbon to the electrode was uneven over the face length. Analysis of the recorded current and voltage waveforms shows that short-circuits and arcing discharges occurred (Figure 5b). Unstable discharges caused the concentration of debris in the gap. Local changes in dielectric resistance and gap size between the electrodes increase the occurrence of arcing and short-circuit discharges (Figure 5b). During the discharge plasma channel reach to 15,000 K [57], significantly above the melting point of graphite [58,59] causing its sublimation. The unstable discharges can have a significant impact on tool wear. Arcing discharges leads to short circuit gap which provide to increase the discharge energy and finally increases tool wear [60]. A similar result was obtained by Li et al. [61]. Nevertheless, in all investigated cases of rough-slot EDM with the graphite electrode in HTCS, we observed significant tool wear (Figure 7b). For this reason, further research into the optimization of the machining process was focused only on the copper electrode.

### 3.2. Response Surface Methodology

Experimental studies were conducted using the Hartley experiment design methodology 5 levels and 2 parameters. According to the adopted design plan, 10 samples with one additional replication in the center point were manufactured and measured. In order to verify the reproducibility of the obtained test results, each experimental trial was repeated. The average results of the experimental studies are presented in Table 5. The surface roughness Ra was in the range of 10.66–16.82 µm. The slot width was in the range of 1.46–1.64 mm. The material removal rate was in the range of 10.41–85.38 mm^3^/min, and the tool wear rate was in the range of 0.11–0.62 mm. 

The response function was built based on the regression analysis and analysis of variance (ANOVA). For the adopted model of regression function (polynomial of the second degree) at a confidence level of 95%, each independent variable was established. ANOVA results after the elimination of non-significant variables for surface roughness Ra, slot width, MRR, and TW are presented in Table 6, Table 7, Table 8 and Table 9, respectively.

Table 6 shows the ANOVA results for surface roughness Ra. The calculated contribution indicates that the discharge current had the highest influence on the surface roughness Ra (90.1%). The second-most affecting variable was the interaction of discharge current with pulse-on-time (5%). The ANOVA results presented in Table 7 indicate that the highest influence on the slot width was pulse-on-time (51.06%), followed by discharge current (22.7%) and square pulse-on-time (19.15%). The contributions of other variables on S were significant but less important. Table 8 presents the ANOVA results for MRR. The obtained results indicate that the square pulse-on-time (71.08%) had the most influence on MRR, followed by the discharge current (13.52%) and pulse-on-time (9.27%). ANOVA results for TW (Table 9) indicate that the highest influence on tool wear had discharge current (76.15%) followed by pulse-on-time (20.76%). 

From the presented ANOVA Table 6, Table 7, Table 8 and Table 9, it can be noted that the models Ra, S, MRR, and TW had Fisher coefficients with values of 19.49, 7.05, 7.25, and 8.38, respectively. The obtained results imply that all developed models were significant at the 95% confidence level.

The response function was built using regression analysis and ANOVA with the backward elimination process. The calculated determination coefficient R^2^ and adjusted coefficient of determination R-Adj for all developed models for surface roughness Ra, the slot width S, MRR, and TW were over 94% and 96%, respectively. These results indicate that the developed regression function has a good adjustment to the test results.

Adopted criteria of ANOVA and regression calculations included the developed function without the non-significant factors. The final version of the response function for the roughness Ra, slot width S, MRR, and TW are described by the following polynomial functions:

Ra = 3.51 + 0.0000012 *t*_on_^2^ + 0.89 *I* − 0.0095 *I*^2^
* − 0.00009 I t*_on_ (6)

S = 0.014 + 0.0054 *t*_on_*^2^* − 0.0065 *I* + 0.002 *I t*_on_^2^ (7)

MRR = −139.1 − 7 − 0.0002 *t*_on_^2^ + 25.627 − *I* − 0.87 *I*^2^ + 0.012 *I t*_on_ (8)

TW = 0.5 − 4 − 0.00035 *t*_on_ + 0.114 *I* − 0.0026 *I*^2^ (9)

**Figure 8:**
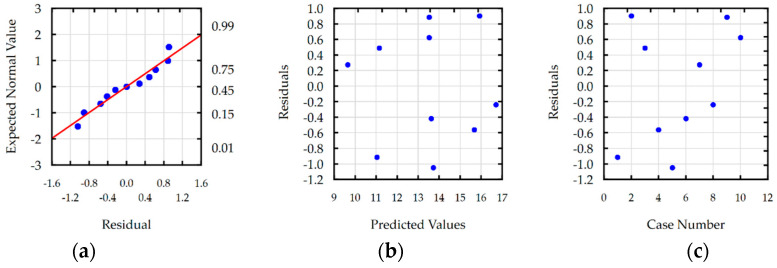
Plots for the model of surface roughness: (**a**) the normal plot of residuals; (**b**) the residuals versus the predicted values; and (**c**) the residuals versus the case number.

**Figure 9:**
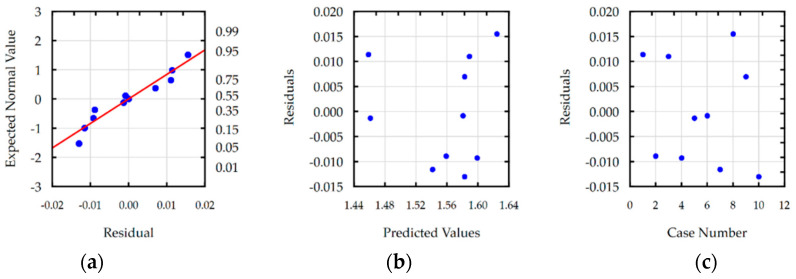
Plots for the model of slot width s: (**a**) the normal plot of residuals; (**b**) the residuals versus the predicted values; and (**c**) the residuals versus the case number.

**Figure 10:**
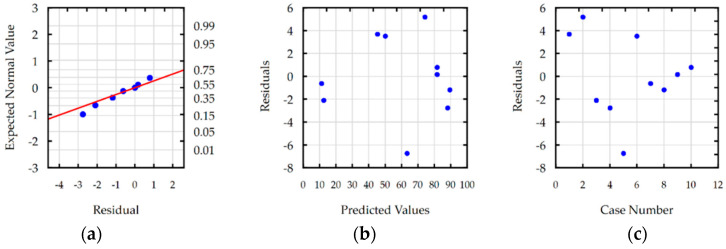
Plots for the model of MRR: (**a**) the normal plot of residuals; (**b**) the residuals versus the predicted values; and (**c**) the residuals versus the case number.

**Figure 11:**
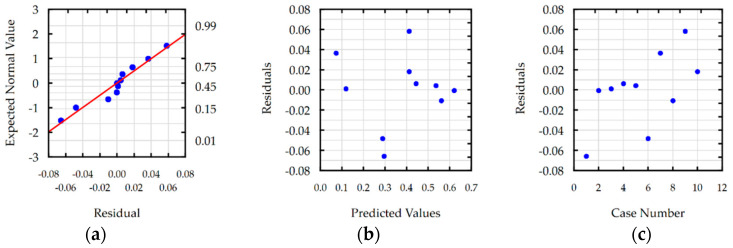
Plots for the model of TW: (**a**) the normal plot of residuals; (**b**) the residuals versus the predicted values; and (**c**) the residuals versus the case number.

Residual analyses checked the response functions for Ra, S, MRR and TW. For each developed function, the normal plot of residuals, residuals versus predicted values, and residuals versus the case number were analyzed. Analysis of residual normal probability plots (Figure 8a, Figure 9a, Figure 10a and Figure 11a) showed that the residuals were normally distributed. Plots of the residuals versus the predicted values (Figure 8b, Figure 9b, Figure 10b and Figure 11b) and the residuals versus the case number values (Figure 8c, Figure 9c, Figure 10c and Figure 11c) indicated that the residuals had a stochastic nature. The analysis of residuals versus the case number indicated that the error terms were independent. Conducted analyses of residuals confirmed that developed models do not reveal inadequacy.

**Figure 12:**
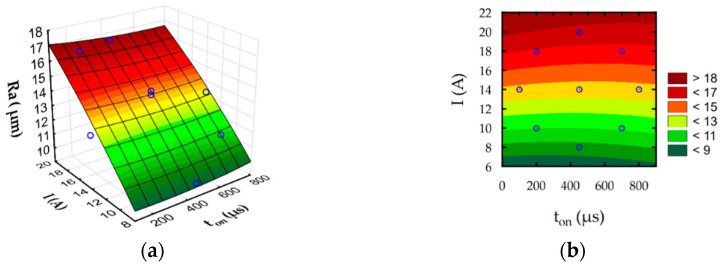
Estimated for roughness Ra: (**a**) response surface and (**b**) contour plot.

**Figure 13:**
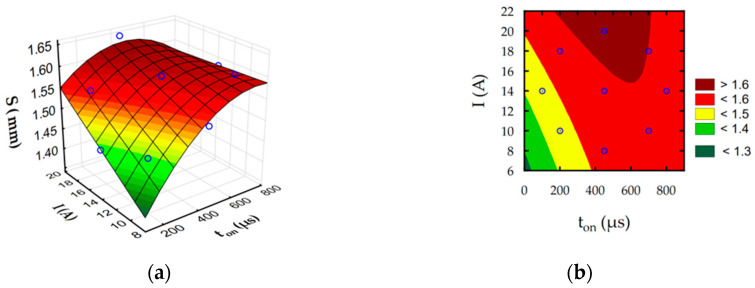
Estimated for slot width S: (**a**) response surface and (**b**) contour plot.

**Figure 14:**
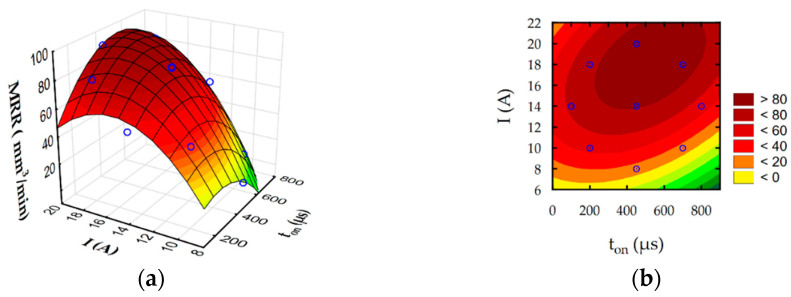
Estimated for MRR: (**a**) response surface and (**b**) contour plot.

**Figure 15:**
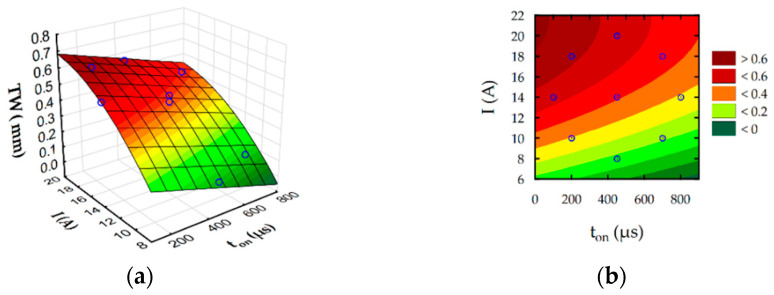
Estimated for TW: (**a**) response surface and (**b**) contour plot.

The developed regression Equations (6)–(9) allow to predict the influence of discharge intensity and pulse-on-time on surface roughness Ra, slot width S, MRR, and TW. Furthermore, estimated response surface plots (Figure 12, Figure 13, Figure 14 and Figure 15) show the relationship between EDM parameters and each independent factor.

Analysis of developed regression models and their graphical interpretation (Figure 12) indicate that the discharge current has a main contribution to the surface roughness Ra during rough-slot EDM. Surface roughness Ra grows with an increase in the intensity of discharge. Furthermore, surface roughness Ra indirectly describes the roughness depth and hence the crater depth. No significant changes in roughness values for the lowest current values with increasing pulse indicate that pulse-on-time does not cause significant changes in the crater depth, although the pulse energy increases significantly. It can be explained by the Gaussian distribution of heat flux in the plasma channel with depends on discharge current and time. The temperature in plasma channel is highest on center of and decrease on the spark radius following normal distribution. Considering this effect increasing the discharge current leads to increase heat flux density [62,63]. For a higher value of discharge intensity, a crater with a larger depth is generated [64]. The amount of removed material during discharge also affected the MRR (Figure 14). Furthermore, the contribution of discharge current on the electrode wear is higher than the pulse-on-time (Figure 15). The volume of removed material, both from the anode and the cathode, mainly depended on the discharge current. The total frequency of discharges depends on the value of pulse-on-time. Grows in pulse-on-time lead to a decrease in frequency. The lower number of discharges affecting the working surface of the electrode leads to a reduction of tool wear. The presented results coincide with the test results of Ayesta et al. [65] during slot machining of 1023 aeronautical alloy.

The complex physics mechanism of material removal process in EDM affected on quality effect of process which can be described by surface layers properties, roughness and geometry accuracy. Increase in intensity and pulse-on-time provides to increase in the slot width (Figure 13). The amount of molten material that was throughout to the gap during discharge and re-solidified increase with the increasing discharge energy. Part of this material re-solidified on the sidewall of electrode (Figure 6). This can lead to discharges occurring not only on the top face of the electrode but also on the surface side of the electrode. The probability of occurrence of electrical discharges at the side of the electrode and workpieces grows. Furthermore, it causes an increase in the sloth width of the manufacturing cavity.

### 3.3. Multi Response Optimization

The Derringer’s desirability was developed for response function (the surface roughness Ra, slot width S, MRR, and TW) (Equations (6)–(9)). A multi-response optimization procedure with global desirability was performed. The adopted methodology includes considering the importance of the estimated value of the response function being close to the minimum or maximum. Constraints and factor ranges for optimization are presented in Table 10.

**Figure 16:**
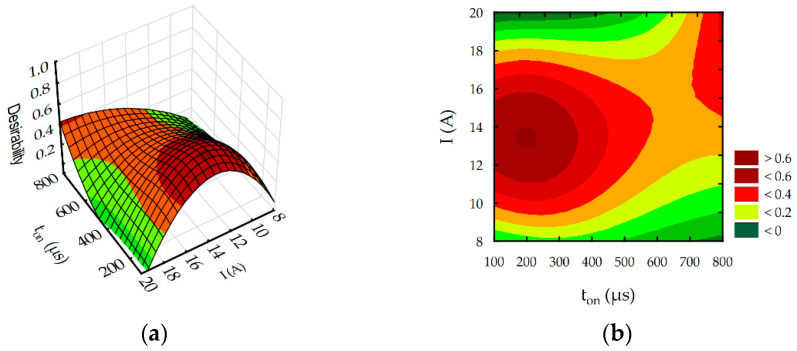
Desirability function for rough-slot EDM optimization: (**a**) response surface and (**b**) contour plots.

**Figure 17:**
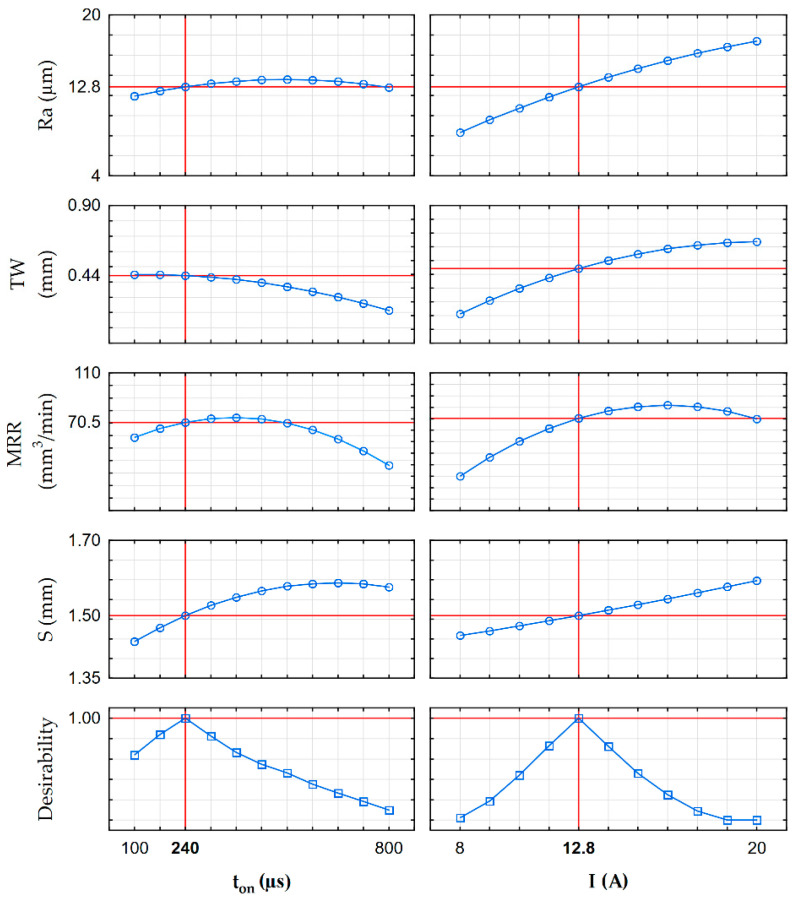
Profiles for predicted values and desirability for adopted criteria of EDM optimization.

The estimated global desirability function for rough-slot EDM of HTCS 150 is presented in Figure 16 and Figure 17. The desirability function reached a value of 1 (Figure 17). The established optimal EDM parameters for rough slotting HTCS 150 were discharge current *I* = 12.8 A and pulse-on-time *t*_on_ = 240 µs. For developed optimal EDM parameters, the predicted TW reached 70.5 mm^3^/min, approximately 20% less than the maximum TW in experimental studies. Predicted surface roughness, slot width, and tool wear rate were Ra = 12.85 µm, S = 1.5 mm, and TW = 0.44 mm, respectively. The obtained results for Ra, TW, and S were less than the maximal values in experimental studies by 30%, 25%, and 9%, respectively.

A confirmation test was performed to check the error between the predicted results of optimization and observed values. Three additional samples were manufactured using the established optimal EDM parameters for the rough slotting of HTCS 150. Machining conditions and average results of tests are presented in Table 11. Analysis of validation results indicates that errors between predictions and experimental results do not exceed 7%. For the adopted optimization criteria, the compromise between material removal rate and values of surface roughness, slot width, and tool wear was achieved.

## 4. Conclusions

In the present work, the main attention was focused on the analytical and experimental investigation of the material removal process during the rough-slot EDM in HTCS with a thin-walled electrode. The influence of pulse-on-time and discharge current on the tool wear, the surface roughness Ra, slot width S, and MRR in High-Thermal-Conductivity Tool Steel was described and established. Furthermore, analyses of wear of two different electrode materials: copper and graphite were provided. In the final stage of research, multi response optimization with the Derringers function was conducted. The following conclusions were drawn:

- The carbon deposition of the dielectric causes diffusion of carbon to the electrode surface. In the analyzed case of rough-slot EDM of HTCS, the diffusion of carbon to the graphite electrode was uneven over the face length. The analysis of registered current voltage waveforms during manufacturing with graphite electrode pointed out occurs as a result of unstable discharges, arcing and short circuit discharges, which lead to increase in wear of the graphite electrode. However, the diffusion of carbon on the copper electrode provided an additional thermal barrier which reduced electrode wear;
- Re-solidified on the sidewall of electrode material, decrease the distance in the gap between the side wall electrode and slot cavity. The probability of occurrence of electrical discharges at the side of the electrode and workpieces grows. Furthermore, this can lead to an increase in the slot width of the manufacturing cavity;
- Discharge current has the main contribution on the surface roughness Ra, slot width S, MRR, and TW during rough-slot EDM with a thin-walled electrode followed by pulse-on-time;
- The global Derringer’s desirability was performed to establish optimal parameters for rough-slot EDM in HTCS. Validation results of multi-response optimization indicate that errors between predictions and experimental results do not exceed 7%;
- The developed predictive models based on regression equations for the rough slotting of highly conductive tool steel can be applied in the build of technological tables of the investigated process and can be applied in modern EDM machines.

## Figures and Tables

**Table 1:** Chemical composition of Rovalma HTCS 150 (mass%).

C	Si	Mn	P	S	Mo	W	Cr	V	Ni	Ti	Cu	Fe
0.31	0.17	0.16	<0.005	<0.003	3.1	1.85	0.08	<0.003	0.08	<0.003	0.1	Bal.

**Table 2:** Physical properties of electrodes [48].

	Particle Size (µm)	Thermal Conductivity (W/mK)	Electrical Resistivity (Ωm)
POCO EDM-3	5	95	1.5 × 10^−5^
Cooper	–	400	1.6 × 10^−8^

**Table 3:** Machining conditions.

Electrode	Copper, Graphite POCO EDM 3
Workpiece material	HTCS 150
Discharge current *I* (A)	8–20
Open voltage U_0_ (V)	225
Discharge voltage (V)	25
pulse-on-time *t*_on_ (μs)	100–800
time interval *t*_off_ (μs)	0.3 *t*_on_
Dielectric	EDM fluid 108 MP-Sin E 60

**Table 4:** Levels of parameters for Hartley design.

EDM Parameters	Level 1	Level 2	Level 3	Level 4	Level 5
Discharge current *I* (A)	8	10	14	18	20
Pulse-on-time *t*_on_ (μs)	100	200	450	700	800

**Table 5:** Design of the experimental matrix with the results of experimental studies.

Exp. No.	EDM Parameters		Observed Values			
	Discharge Current *I* (A)	Pulse-on-Time *t*_on_ (μs)	Surface Roughness Ra (μm)	Slot Width S (mm)	TW (mm)	MRR (mm^3^/min)
1.	10	200	10.66	1.47	0.23	49.06
2.	18	200	16.82	1.55	0.62	79.34
3.	10	700	13.84	1.6	0.12	10.41
4.	18	700	12.42	1.59	0.45	85.38
5.	14	100	13.07	1.46	0.54	56.65
6.	14	800	11.94	1.58	0.24	53.71
7.	8	450	10.72	1.53	0.11	10.55
8.	20	450	15.55	1.64	0.55	88.3
9.	14	450	13.60	1.59	0.47	81.82
10.	14	450	13.31	1.57	0.44	82.44

**Table 6:** ANOVA table for Ra (after elimination).

Source	Sum of Squares	Degrees of Freedom	Mean Square	*F*-Value	Prob > *f*	Contribution %
Model	52.1256	4	13.0314	19.49	0.0029	
*t*_on_^2^	1.5672	1	1.5672	11.72	<0.0001	3.01
*I*	46.9647	1	46.9647	351.26	0.0004	90.10
*I* ^2^	0.9855	1	0.9855	7.37	0.0420	1.89
*t*_on_ *I*	2.6082	1	2.6082	19.50	0.0069	5.00
Error	0.6685	5				
Total SS	52.7941	9	*R-sqr =* 0.98		*R-Adj =* 0.97	

**Table 7:** ANOVA table for slot width S (after elimination).

Source	Sum of Squares	Degrees of Freedom	Mean Square	*F*-Value	Prob > *f*	Contribution %
Model	0.0282	4	0.0070	7.05	0.0274	
*t*_on_	0.0144	1	0.0144	71.31	0.0003	51.06
*t*_on_^2^	0.0054	1	0.0054	26.70	0.0035	19.15
*I*	0.0064	1	0.0064	32.12	0.0023	22.70
*t*_on_ *I*	0.0020	1	0.0020	10.01	0.0249	7.09
Error	0.0010	5				
Total SS	0.0292	9	*R-sqr =* 0.96		*R-Adj =* 0.94	

**Table 8:** ANOVA table for MRR (after elimination).

Source	Sum of Squares	Degrees of Freedom	Mean Square	*F*-Value	Prob > *f*	Contribution %
Model	8147.74	4	2036.93	7.25	0.0259	
*t*_on_	754.90	1	0.0144	71.31	0.0144	9.27
*I*	5791.59	1	0.0054	26.70	0.0001	71.08
*I* ^2^	1101.96	1	0.0064	32.12	0.0068	13.52
*t*_on_ *I*	499.29	1	0.0020	10.01	0.03074	6.13
Error	280.81	5				
Total SS	8428.55	9	*R-sqr =* 0.96		*R-Adj =* 0.94	

**Table 9:** ANOVA table for tool wear rate TW (after elimination).

Source	Sum of Squares	Degrees of Freedom	Mean Square	*F*-Value	Prob > *f*	Contribution %
Model	0.2981	3	0.0993	8.38	0.0144	
*t*_on_	0.06186	1	1.5672	11.72	<0.0001	20.76
*I*	0.2240	1	46.9647	351.26	0.0004	75.15
*I* ^2^	0.0122	1	0.9855	7.37	0.0420	4.09
Error	0.0118	6				
Total SS	0.3099	9	*R-sqr =* 0.96		*R-Adj =* 0.94	

**Table 10:** Goals and factor range for optimization.

Factors	Goal	Lower Limit	Upper Limit	Weight	Importance
*I* (A)	In range	8	20		-
*t*_on_ (µs)	In range	100	800		-
Ra (µm)	Minimize	10.66	16.82	1	*p* = 0.3
S (mm)	Minimize	1.46	1.64	1	*p* = 0.3
MRR (mm^3^/min)	Maximize	10.41	85.38	1	*s* = 5
TW (mm)	Minimize	0.11	0.62	1	*p* = 0.3

**Table 11:** Experimental validation of the values obtained from multi-response optimization.

Optimal EDM Parameters	Summary of Values Obtained in Optimization			
	Response	Predicted	Experimental Verification	Error %
*I* = 12.8 A	Ra (µm)	12.85	12.1	7
*t*_on_ = 240 µs	S (mm)	1.5	1.45	4
*t*_off_ = 0.3 *t*_on_	MRR (mm^3^/min)	70.5	67	5
*U*_c_ = 25 V	TW (mm)	0.44	0.41	6

## Data Availability

The data presented in this study are available on request from the corresponding authors. The data are not publicly available due to privacy.
